# A Rare Case of Vulval Leiomyoma

**DOI:** 10.7759/cureus.67141

**Published:** 2024-08-18

**Authors:** Prajakta Ghewade, Sunita Vagha, Pravin Gadkari

**Affiliations:** 1 Pathology, Jawaharlal Nehru Medical College, Wardha, IND

**Keywords:** smooth muscle tumour, benign, rare, leiomyoma, vulva

## Abstract

Vulval leiomyomas are exceedingly rare benign smooth muscle tumours, with limited cases reported in the literature. They often present a diagnostic challenge due to their uncommon location and nonspecific clinical presentation, mimicking other vulval pathologies. We present the case of a 40-year-old female who reported a painless, slowly enlarging mass on the left side of the vulva. An excisional biopsy was performed, and histopathological analysis confirmed the diagnosis of vulval leiomyoma. Vulval leiomyomas are rare and may be misdiagnosed due to their similarity to other vulval lesions such as Bartholin cysts, lipomas, or fibromas. The standard treatment is surgical excision, which is often curative.

## Introduction

Leiomyomas are benign, well-circumscribed soft tissue tumours that originate from mesenchymal cells. Leiomyomas account for 3.8% of all benign soft tissue tumours [1]. Leiomyomas, atypical leiomyomas, and leiomyosarcomas are the three main categories for vulva smooth muscle tumours. Dartos muscle, erectile tissue, and smooth muscle within the round ligament are the origins of vulvar leiomyomas [1]. While leiomyomas can arise anywhere where a smooth muscle is present, the uterus is the site of the majority of the cases. The extremely rare nature of vulva smooth muscle tumours may cause them to be mistakenly diagnosed as benign conditions such as abscesses or Bartholin cysts [2]. Just 0.03% of gynaecologic neoplasms and 0.07% of vulvar tumours are vulvar leiomyomas [1]. Depending on their size and location, vulval leiomyomas can occasionally produce discomfort, pain, or pressure in addition to being typically slow-growing, painless masses [3,4]. Although the exact aetiology of vulval leiomyomas is unknown, hormonal variables that are similar to those found in uterine leiomyomas may be involved. Due to the small number of instances reported in the literature, vulval leiomyomas are rare, which usually causes diagnostic ambiguity. The confirmation of the diagnosis is often achieved by histopathological study. Tissue analysis is needed for a definitive diagnosis, even though imaging modalities like ultrasonography or MRI can aid in evaluating the lesion. Surgical excision is frequently used to treat vulval leiomyomas, and it is usually curative. However, given their rarity and risk of mistaken diagnosis, practitioners must have a full awareness of this disorder. This case report aims to highlight a rare presentation of vulval leiomyoma, emphasizing the diagnostic challenges and management strategies to enhance awareness among healthcare providers.

## Case presentation

**Figure 1:**
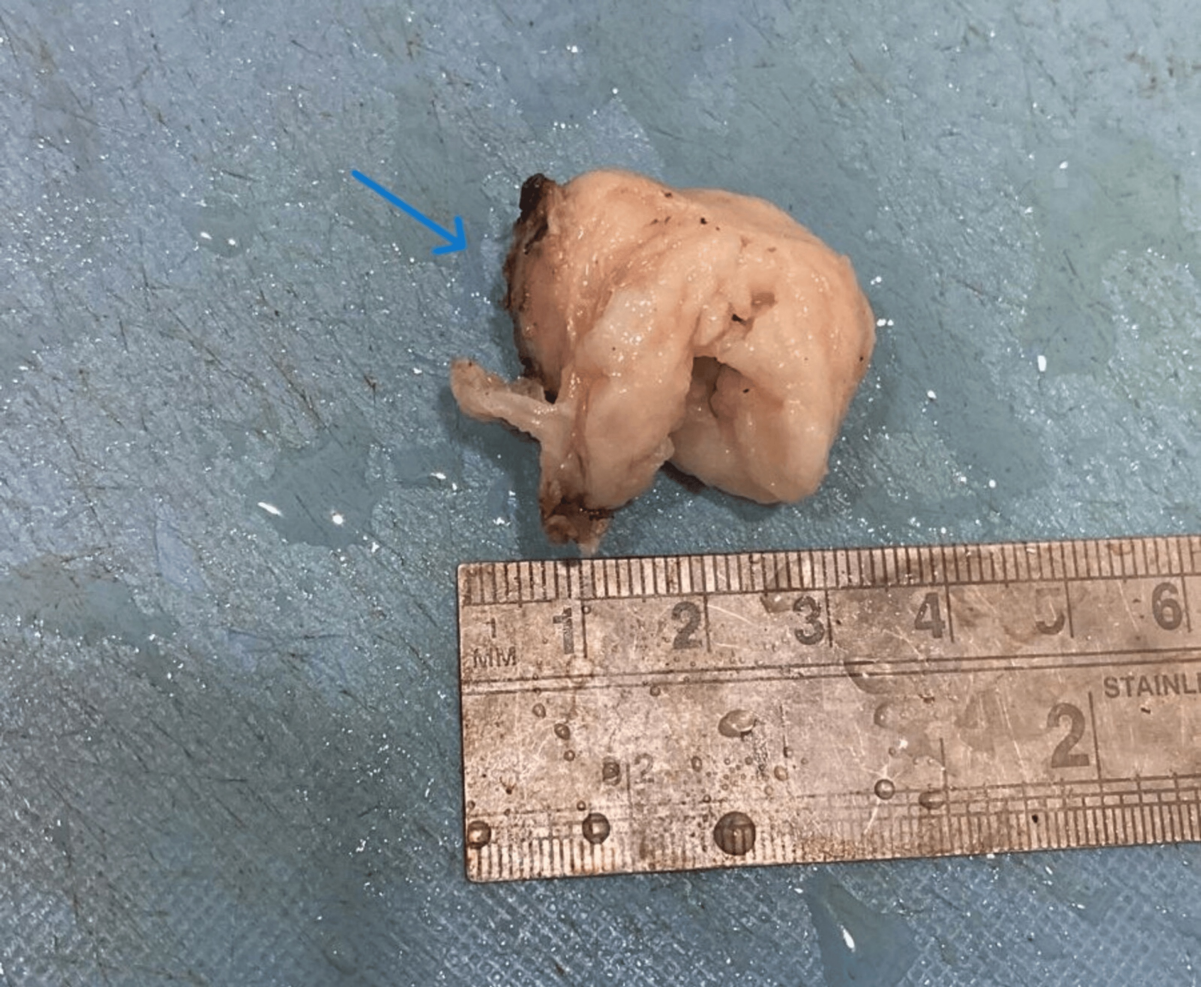
Gross image of specimen received in the histopathology lab (blue arrow).

A 40-year-old woman came to the outpatient department with complaints of a painless swelling on the left side of her vulva, which was gradually growing in size. The patient reported no other symptoms, including blood, discharge, or changes in urination patterns, aside from slight discomfort. A solid, well-circumscribed, non-tender lump with a diameter of around 3 x 2 cm was found upon physical examination. Complete blood count showed haemoglobin levels of 11.2 g/dl, white blood cell count of 4,500 cells/cu.mm and platelet count of 4,72,000 cells/cu.mm. Pelvic ultrasonography showed a firm, hypoechoic mass that was compatible with a benign lesion. The differential diagnosis considered were Bartholin cyst, myoma and lipoma. An excisional biopsy was done and the specimen was sent to the histopathology lab for examination. Grossly, we received a single, whitish, firm mass measuring 3.5 x 3 x 1.8 cm (Figure 1). 

**Figure 2:**
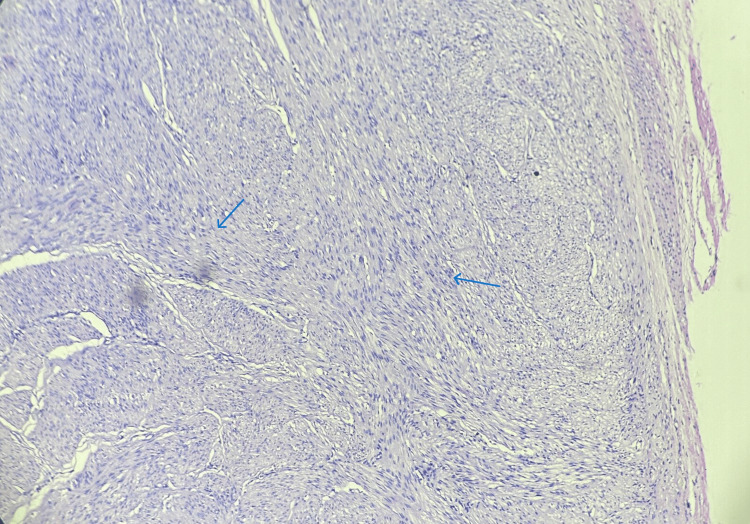
Histopathology image of vulval leiomyoma showing interlacing bundles of smooth muscle cells devoid of atypia or mitotic activity (10x magnification, blue arrow)

**Figure 3:**
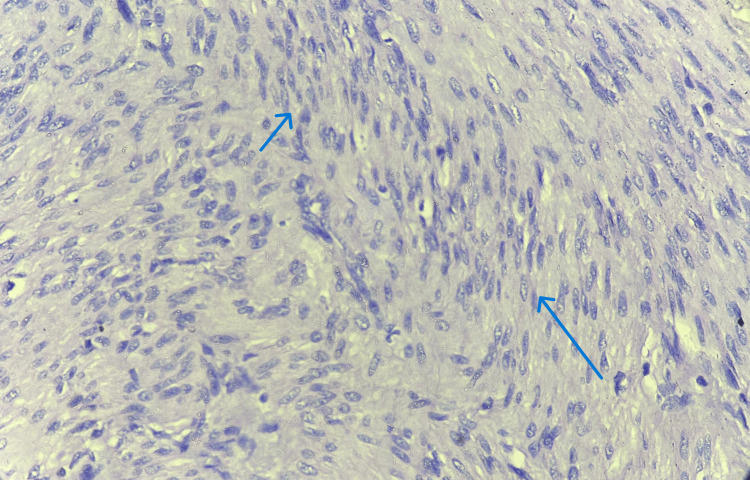
Histopathology image of vulval leiomyoma showing interlacing bundles of smooth muscle cells devoid of atypia or mitotic activity (40x magnification, blue arrow)

Histopathological examination of sections stained with haematoxylin and eosin (H&E) confirmed the diagnosis of vulval leiomyoma, which is characterized by interlacing bundles of smooth muscle cells devoid of atypia or mitotic activity (Figures 2, 3). 

Post-operatively, the patient was managed with antibiotics and supportive treatment. She was called for follow-up after seven days. The patient is now completely cured without any recurrence.

## Discussion

Vulval leiomyomas, which make up 0.03% of all gynaecological tumours and 0.07% of all vulvar neoplasms, are incredibly uncommon benign tumours that originate from smooth muscle cells in the vulva [1]. Due to the tumour's uncommon nature and vague appearance, diagnosis is frequently difficult. This 40-year-old woman's case of vulval leiomyoma brings to light a number of crucial factors to take into account when diagnosing and treating this uncommon ailment. Vulval leiomyomas can appear as painless, slowly expanding lumps. Depending on their size and location, they may occasionally result in pain, discomfort or dyspareunia [5]. A vulval lump can have a wide range of differential diagnoses, including lipomas, sebaceous cysts, Bartholin's gland cysts and other benign or malignant tumours [6]. A meticulous and in-depth investigation is required due to this overlapping clinical appearance. In this instance, the patient had a hard, painless lump in the vulva that had been growing bigger over time. A benign process was first suggested by the lack of acute symptoms, such as excruciating pain or ulceration; however, a malignant process could not be ruled out without additional research.

**Table 1:** Review of cases of vulval leiomyoma reported in past literature.

Author	Age	Clinical signs and symptoms	Size	Histopathological diagnosis	Treatment
Pandey et al. 2014 [7]	20	Painful swelling in vulva	6 x 4 x 3 cm	Epithelioid leiomyoma	Surgical excision
Zhao et al. 2015 [8]	30	Vulvar mass	7 cm	Epithelioid leiomyoma with myxoid stroma	Surgical excision
Sui et al. 2016 [9]	60	Painful perineal mass	-	Perineal leiomyoma	Surgical excision
Ammouri et al. 2019 [10]	40	Vulvar mass	5 cm	Vulval leiomyoma	Surgical excision
Jang et al. 2019 [11]	45	Painful vulvar mass on right labia minora	1.7 x 1.4 cm	Vulvar epithelioid leiomyoma	Excisional biopsy
Fontinele et al. 2021 [12]	14	Tumour in right hemivulva	10 x 10 cm	Vulval leiomyoma	Surgical excision
Ashraf Muhammed et al. 2022 [13]	41	Left vulvar lump	5 x 2 cm	Benign vulvar leiomyoma	Surgical excision
Goyal et al. 2023 [14]	15	Painless swelling in left labia majora	14 x 10 cm	Benign vulvar leiomyoma	Surgical excision
Komatineni et al. 2023 [6]	44	Painless vulval tumour	5 x 5 x 4 cm	Vulval leiomyoma	Incision and repair
Anurag et al. 2023 [15]	Middle aged	Left side of labia majora	10.5 x 4 cm	Vulvar leiomyoma with secondary degeneration	Surgical excision

Table 1 summarizes the findings of a few cases of vulval leiomyoma that were reported in the past literature. All these patients presented with a painless vulval mass, variable in size. Surgical excision was done and histopathological analysis confirmed the diagnosis of vulvar leiomyoma. The findings in all these cases were similar to the findings in our case. Imaging studies, particularly ultrasonography, can reveal important details about the nature of vulval masses. In this case, pelvic ultrasonography revealed a well-defined, hypoechoic lesion, indicating a benign tumour. However, imaging alone cannot differentiate leiomyomas from other vulval lesions, emphasizing the significance of histological testing [15] . Histopathology is the gold standard of diagnosis [14]. Microscopically, the tumour is comprised of intertwined bundles of smooth muscle cells, with varying degrees of well-vascularized connective tissue separating them [16]. In this case, an excisional biopsy confirmed the diagnosis of vulval leiomyoma, which has the typical histological characteristics of this benign tumour. Surgical excision is frequently used to treat vulval leiomyomas, and it is usually curative [1]. Complete excision is necessary to alleviate symptoms and avoid recurrence. Given the region's cosmetic and functional importance, the surgical technique should try to preserve its vulval structure and function. In this case, the surgical excision was effective, and the patient reported great symptom improvement following surgery. There was no evidence of recurrence during follow-up, which is consistent with the good prognosis associated with full excision of leiomyomas. Though their specific aetiology is uncertain, pluripotent mesenchymal cells that develop into smooth muscle cells are thought to be the source of vulval leiomyomas [17]. Hormonal effects may play a role, as estrogen and progesterone receptors have been found in some leiomyomas, which are similar to uterine fibroids [1]. Although uncommon, knowledge of this entity can aid in quick diagnosis and care, avoiding unneeded interventions or concern for the patient. When assessing vulval masses, clinicians should keep a high suspicion for benign tumours such as leiomyomas, especially if clinical characteristics point to a non-malignant cause. A histopathological study should be performed to confirm the diagnosis and suggest the proper treatment.

## Conclusions

Leiomyomas are typical benign tumours that can affect multiple organs in the body. While they are most common in the uterus, they can also appear on the skin, gastrointestinal tract and vulva. Understanding the clinical characteristics, diagnostic procedures and therapy options is critical for their optimal management and enhancing the quality of life for those affected. Early detection and targeted treatment methods can assist in alleviating the symptoms and address patient-specific needs. More research into the pathophysiology and epidemiology of vulval leiomyomas is needed to improve our understanding of this uncommon condition. Data gathered through case reports and series can provide useful insights into the clinical behaviour, best care techniques and long-term results of vulval leiomyomas.
